# Agronomic Management and Rice Varieties Controlling Cd Bioaccumulation in Rice

**DOI:** 10.3390/ijerph16132376

**Published:** 2019-07-04

**Authors:** Liangmei Chen, Wenge Wu, Fengxiang Han, Jiangxia Li, Wenling Ye, Huanhuan Fu, Yonghua Yan, Youhua Ma, Qiang Wang

**Affiliations:** 1 College of Resources and Environmental Sciences, Anhui Agriculture University, Hefei 230001, China; 2 Department of Chemistry and Biochemistry, Jackson State University, Jackson, MS 39056, USA; 3 Rice Research Institute, Anhui Academy of Agricultural Sciences, Hefei 230031, China; 4 Department of Math, Jackson State University, Jackson, MS 39056, USA

**Keywords:** rice, varieties, Cd, biological organic fertilizers, lime, porous ceramic nanomaterials

## Abstract

Selection of rice varieties and application of amendments are effective measures to ensure food safety. Here we report that in the non-Cd area, the grain quality of all rice varieties met the Chinese National Grain Safety Standards (CNGSS). In the high-Cd area, rice varieties showed significant different bioaccumulation of Cd with lower rice yields than those in non-Cd area with the average decrease of 31.1%. There was a negative correlation between grain Cd content and yields. A total of 19 rice varieties were selected as low Cd accumulating rice varieties and their Cd content met CNGSS in the low-Cd area. Six of them met CNGSS in the high-Cd area. The application of amendments significantly reduced Cd content in rice grains by 1.0–84.7% with an average of 52.6% and 13 of varieties met CNGSS. The amendments reduced available Cd content in soils by 1.1–75.8% but had no significant effects on rice yields. Therefore, the current study implied that proper agronomic management with selection of rice varieties and soil amendments was essential in controlling Cd accumulation in rice grains.

## 1. Introduction

Rice is one of the main food crops in the world. However, it is capable of absorbing toxic heavy metals such as cadmium. Cadmium can be readily accumulated in rice, resulting in Cd content in grains exceeding the human consumption standard limits. This became an increasingly serious food safety concern, especially in developing countries with fast economy development [1,2]. High Cd concentration in plants retards their growth and development, producing poor grains and low yields. Further enrichment in food chain eventually causes serious Cd threat to human and animal health. To produce high-yield rice with superior quality, it is necessary to take a series of comprehensive management and agronomic practices in reducing rice Cd content.

Screening low Cd accumulating varieties, agronomic practices as well as remediation techniques are useful methods to reduce Cd accumulation in rice plants, improve rice quality, and prevent Cd from entering food chains. Low accumulating varieties were utilized successfully in bread wheats, sunflower, and other crops [3,4]. Previous studies identified some genetic variation in heavy metal uptake and their deposits in grains. Liu et al. observed that rice varieties varied greatly both in absorption of Cd and in responses to Cd [5]. Shi et al. tested 110 rice varieties in fields and observed that Cd concentrations of super rice varieties were 14 times that of hybrid rice [6]. Yu et al. also proved that there were significant differences among rice varieties and 30 out of 43 cultivars were tested as relatively safe varieties regarding Cd accumulation [7]. Liu et al. found that Cd concentrations ranged from 0.22 to 2.86 mg kg^−1^ in brown rice among cultivating 52 rice varieties.^5^ It was shown to be feasible to screen rice varieties with low accumulation of cadmium as the means to control heavy metal bioaccumulation [8,9]. Furthermore, previous studies showed that the application of biological organic fertilizers not only reduced contents of soil available heavy metals, but also improved soil fertility leading to high crop economic yields [10,11]. Lime reduced diethylene triamine pentacetic acid (DTPA) extracted heavy metal content through raising soil pH [12]. SAMMNS porous ceramics are a new type nanocomposite material with unique highly self-assembly and high density functional single molecules and controllable ordered high porosity. The materials had a large, selective adsorption capacity, and long lifetime [13]. Combining these materials not only reduced the effective cadmium content in the soil, but also improved the soil environment. To effectively prevent Cd accumulation in foods, the best strategy is to combine low Cd rice varieties with soil remediation technology [14,15].

The objectives of this study were to investigate the effects of rice varieties and agronomic practices on controlling rice Cd accumulations. We proposed the hypothesis that coupling low Cd accumulating rice varieties with soil amendments significantly reduced Cd accumulation in rice grains. A comprehensive strategy for growing rice on Cd contaminated soil was developed. 

## 2. Material and Methods

### 2.1. Description of Field Experimental Plots

#### 2.1.1. Methods for Soil and Plant Sampling

The diagonal sampling method was used to collect soil and plant samples. Three points were selected along diagonally across the rectangular test area as sampling points. Soil was taken from depths of 1–5 cm and 5–15 cm, and mixed well. The plant sample was collected on the aerial part and the sampling point was in one-to-one correspondence with the sampling point of the soil sample. At the same time sufficient sample sizes from each point were ensured. Then the composite plant samples of the three points were mixed.

#### 2.1.2. Test Area Background

We defined a low cadmium region with a soil contaminant content of 1–2 times the screening value (0.3 mg·kg^−1^) of GB15618-2018, and a high cadmium region with a content exceeding the risk intervention values (1.5 mg·kg^−1^) of GB15618-2018 with the single pollution index evaluation method.

The experiments were conducted in a mine area in Tongling, Anhui Province, China. The farmlands were classified into low-Cd area and non-Cd area. Soil Cd background content in the area was 0.196 mg.kg^−1^ (Geological Survey of the Ministry of Environmental Protection) [16]. The soil was red loam. Some selected soil physical and chemical properties, and Cd background levels were described in Table 1.

### 2.2. Rice Varieties and Stabilization Agents

#### 2.2.1. Rice Varieties Used for the Screening Test

For the screening test, the following 45 rice varieties were used.

A. Conventional Japonica Rice Cultivars (19 varieties): 

a) The medium maturity medium Japonica variety (3) including: Xudao5, Xinkedao21, Xudao7;

b) The late maturity medium Japonica varieties (5): Wandao 68, Zhendao 14, Ningjing 4, Yangyujing2, Nanjing49;

c) The early maturity late Japonica varieties (8) including: Ningjing 2, Zhendao 11, Wandao 94, Jiahua 1, Wuyunjing 23, Wuyunjing 29, Yangjing 4227, Zhendao 18;

d) The Mid maturity late Japonica varieties (3): Nanjing 94140, Xiushui 63, Xiushui 134;

B. The Hybrid Japonica rice varieties (3):

a) Medium japonica variety: III you 98;

b) Late japonica varieties: Yongyou 8, Jiayou 2. 

C. Conventional Indian rice varieties (5): 

Teqing, IR36, Nanjing 11, Zhenzhuai, Guichao 2. 

D. Hybrid Indica rice varieties (18):

a) Two-line hybrid Medium Indica rice (15): Xinliangyou 6, Xinliangyouxiang 4, Xinliangyou 106, Feng Liangyou 1, Feng liang you xiang 1, Hui Liangyouc898, Liangyouxin 90, Guangliangyou 1128, Liangyou 6206, Liangyou 378, Liangyou 766, Liangyou 8106, ZhunLiangyou 608, Shenliangyou 1813, Xiangliangyou 2 

b) Three-line hybrid medium Indica rice varieties (3): Ⅱyou 293, F you 498, II you 838

#### 2.2.2. Low Cd Rice Varieties Used for Remediation

For testing the effects of low Cd rice varieties in combination with the mixture of limes, organic fertilizers and SAMMNS porous ceramics nanomaterials on rice Cd contents. The following rice varieties were used: Ningjing 4, Zhendao 11, Xiushui 63, Xudao 5, Xudao 7, Ningjing 5, Jiayou 2, Zhendao 14, Wandao 68, Yangjing 4227, Zhendao 18, Nanjing 11, Fengliangyouxiang 1, Liangyou 6206, Xinliangyou 106, Xinkedao 21, Teqing, Xinliangyou 917, Xinliangyou 1671, Liangyou 766, Zhenzhuai, Liangyou 8106 which were selected from studied conducted during first two years.

A mixture of lime, organic fertilizers, and SAMMNS porous ceramics was used as the soil stabilization agents. 

### 2.3. Rice Variety Screening Experiment Design

Forty-five rice varieties were grown from 2014–2015 for two consecutive years in a high-Cd area and a non-Cd area. The high-Cd area and non-Cd area were two rectangular areas (L×W: 120 m × 30 m) separated by cement concrete, and each area was designed with split-plot experiment design. The distance between two adjacent varieties was 50 cm, and the area of each variety was 6 m^2^ (2 × 3 m). Each variety had three replicates with the total 135 plots. Each of the 45 varieties in each of the three replicates was randomly distributed in a grid (5 x 9). The three repeats are arranged side by side with the middle separated by cement concrete. Rice plants were randomly planted at a space of 20 cm between rows. Direct seeding rate of conventional rice varieties was 50 kg·hm^−2^ and that of hybrid rice varieties was 40 kg·hm^−2^. Fertilizers included fully-fermented manure applied at 14990 kg·hm^−2^ and commercial organic fertilizer at a rate of 1200–1500 kg·hm^−2^. nitrogen fertilizer rate was 225 kg·hm^−2^ for the Japonica rice and 210 kg·hm^−2^ for Indica rice varieties. Phosphate fertilizer (P_2_O_5_) was used at 60 kg·hm^−2^ and potash fertilizer (K_2_O) at 120 kg·hm^−2^. 

There were four stages for fertilization. Therefore, we divided all the nitrogen fertilizer and potash fertilizer that the rice needed into four scale parts. Organic and potash fertilizers were applied as base fertilizers containing 40% nitrogen fertilizer and 60% potash fertilizer and 1kg hm^−2^ ZnSO_4_. Additional fertilizers were used in the rice tillering stage (15% nitrogen fertilizer), panicle (35% nitrogen fertilizer and 40% potash fertilizer), and grain stages (10% nitrogen fertilizer). Seeds were sown in May and crops were harvested at the end of September. Plants and soil samples were collected to analyze Cd contents in grains and soil. Rice yield data were collected after samples. All the rice grain of each variety were collected and weighed separately for yield measurement. 

### 2.4. Soil Remediation Experiments with a Combination of Low Cd Accumulating Rice Variety and Applications of Stabilization Agents 

In 2016, 19 low-Cd-accumulating rice varieties selected from the previous two-year screening experiment were planted in the high-Cd and low-Cd areas. Each rice variety plot was 334 m^2^. The first half of the plot was applied with the mixture of lime (900 kg·hm^−2^)-biological organic fertilizer (3000 kg·hm^−2^) and SAMMNS porous ceramics (3000 kg·hm^−2^) and the other half was blank. The amendment rate values determined by previous soil amendment repair experiments we did before. The method of incorporating the amendments into the soil was as follows. Firstly, before transplanting rice, we apply the required amount of lime to the planting area of each variety, and then plow the soil to evenly stir the lime into the farm soil. Then the bio-organic fertilizer is applied one week after the lime application, and the application method and the tillage method are the same as the lime. Finally, the porous ceramic nanomaterial is applied before the irrigation of the farmland, and the application method and the tillage method are the same as the lime, and then the farmland is irrigated. The direct seeding rate of conventional rice varieties was 50 kg·hm^−2^ and that of hybrid rice varieties was 40 kg·hm^−2^ with a planting distance of 20 cm. Fertilizer application followed the same scheme of the previous two-year screening experiment. 

Biological organic fertilizer was purchased from Beijing Long Age AMMS biological technology Co., Ltd. The colony-forming unit (CFU) of the fertilizer was ≥0.20×10^8·g^−1^ and the organic matter was 20.0% or more. The effective bacterial species were *Bacillus subtilis* and *Paenibacillus mucilaginosus*. The modified porous nanoceramic material from Anhui GFTEM Environmental Protection Science and Technology Co., Ltd. was a new high self-assembled, densely functional single molecule, ordered and controlled porous nanocomposite. This material had functional groups introduced on the pore surface of ceramic nanomaterials (silicon or ceramic as the carrier). It combined submicroporous ceramic material preparation technology and single molecule self-assembly technology. The submicroporous ceramic material was a novel material with a high pore size controllable specific surface area and its pore size can be controlled from 5 nm to 2000 nm with the specific surface area of 900 m^2^·g^−1^, which provides the high adsorption rate necessary for chemical separation. Single-molecule self-assembly technology formed organic layer molecules with functional groups to generate monolayer bonds on the surface of the ceramic in an orderly and high-density manner. This provided submicroporous ceramics with excellent molecular recognition capabilities to effectively remove heavy metals or other target contaminants.

### 2.5. Analytical Methods

Cd content in rice grains was analyzed using the GB/T5009.15-2003 method. About 0.5g brown rice samples were digested with 10 mL HNO_3_ in a CEM MARS6. The contents of the elements were measured with graphite furnace atomic absorption spectrophotometry (Jena Z700P atomic absorption spectrophotometer, Gena, Germany). 

Available Cd content in soil was analyzed with GB/T 23739-2009 method. About 5.0 g soil sample was shaken for 2 h with 25 mL diethylene triamine pentacetic acid on a horizontal reciprocating oscillator. Mixed solution was filtered through filter paper. Cd in the filtrate solutions was measured with graphite furnace atomic absorption spectrophotometry. 

Soil pH and nutrients contents were assayed with the Chinese national standard assay methods (pH: GB 7859-1987; N: GB 7173-1987, P: GB 7853-1987, K: GB 7856-1987; organic matter: GB 9834-1988).

### 2.6. Statistical Analyses

Significance analyses among the treatments (P < 0.05) were conducted using SPSS19.0 (SPSS Inc., Chicago, IL, USA) and Duncan’s new multiple range test. Figures and tables were completed with Origin 8.0 (Originlab, Northampton, MA, USA) and Excel 2007 (Microsoft, Redmond, WA, USA).

## 3. Results 

### 3.1. Cd Accumulation in Rice Varieties

**Figure 1:**
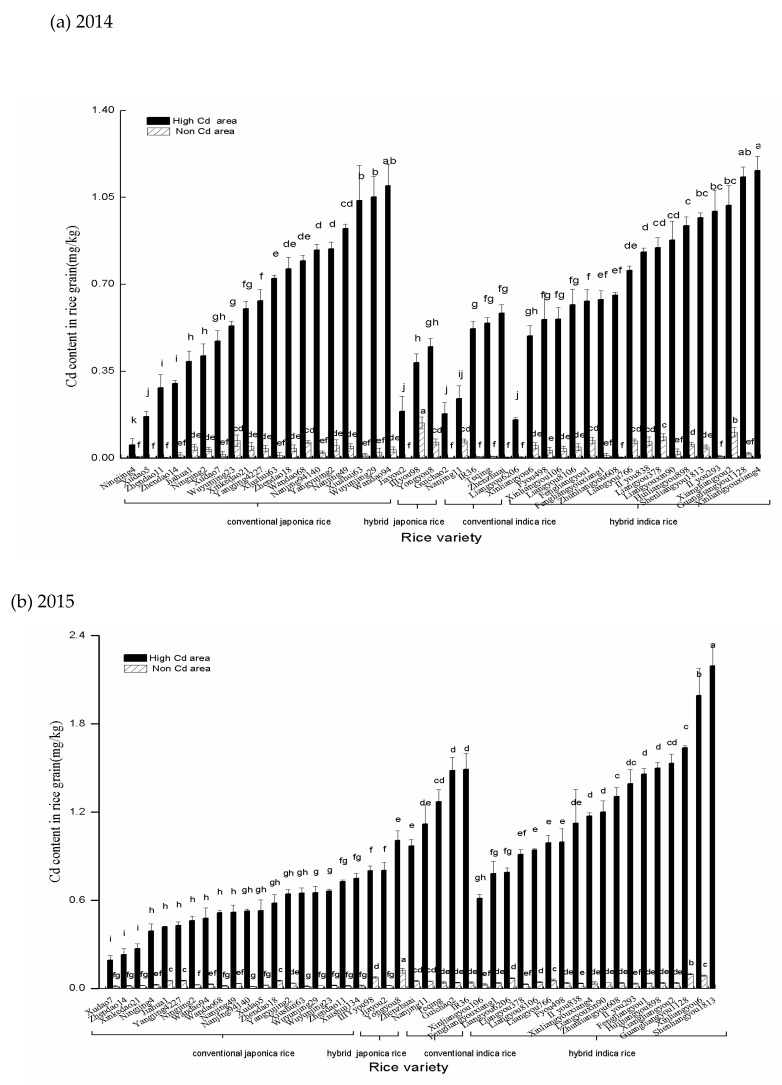
Cd content in rice grains in (**a**) 2014 rice season and (**b**)2015 rice season. Different letters above columns indicate significant difference among varieties at 0.05 level.

Cd contents in rice grains cultivated in 2014 and 2015 were shown in Figure 1. In the first two-year experiments, there were significant differences in grain Cd content among rice varieties (*P* < 0.05). In 2014 and 2015, grain Cd content of rice planted in the non-Cd area was 0.0012-0.14 mg.kg^−1^, which was below the national grain safety standard of 0.2 mg·kg^−1^ as specified in GB2762-2012.

In 2014, the grain Cd content of Japonica rice planted in the high-Cd area was 0.05–1.05 mg·kg^−1^ with an average of 0.59 mg·kg^−1^ and a variance index of 51.9%. Grain Cd content of the following rice varieties (Xudao 5, a medium maturity Japonica variety), Ningjing 4 (a late maturity Japonica variety), Jiayou 2 (a late Japonica variety) reached the national food safety standard. The Cd content in Indica rice grains was 0.16~1.16 mg·kg^−1^ with an average of 0.69 mg·kg^−1^ and a variance index of 40.6%. Grain Cd content of the following rice varieties: Guichao 2, a conventional Indica variety) and Liangyou 6206 (a two-line hybrid medium Indica variety) reached the national food safety standard.

In 2015, the grain Cd content of Japonica rice produced in the high-Cd area was 0.19–1.01 mg·kg^−1^ with an average of 0.56 mg·kg^−1^ and a variance index of 35.8%. The grain Cd content of Xudao 7 (medium maturity medium japonica variety) met the national food safety standard. The grain Cd content of Indica rice was 0.61–2.19 mg·kg^−1^ with an average of 1.26 mg·kg^−1^ and a variance index at 30.4%. None of the Indica rice varieties met the national food safety standard. These results indicated that Japonica rice grain had a lower accumulation of cadmium than the Indica rice, i.e., conventional varieties had a lower accumulation of cadmium than hybrid rice. 

From the above field experiments, the following 19 rice varieties had low Cd accumulation: Ningjing 4, Zhendao 11, Xiushui 63, Xudao 5, Xudao 7, Jiayou 2, Zhendao 14, Wandao 68, Yangjing 4227, Zhendao 18, Nanjing 11, Fengliangyouxiang 1, Liangyou 6206, Xinliangyou 106, Xinkedao 21, Teqing, Liangyou 766, Zhenzhuai, and Liangyou 8106. These varieties may be used as low Cd accumulating varieties in future studies. The Cd sensitive varieties included Liangyouxin 90, Huiliangyou 898, Shenliangyou 1813, Xiangliangyou 2, Guangliangyou 1128, Xinliangyouxiang 4, IIyou 838, and II you 293. These varieties were hybrid rice.

### 3.2. Yields of Rice Varieties and Their Sensitivity to Cd

**Figure 2:**
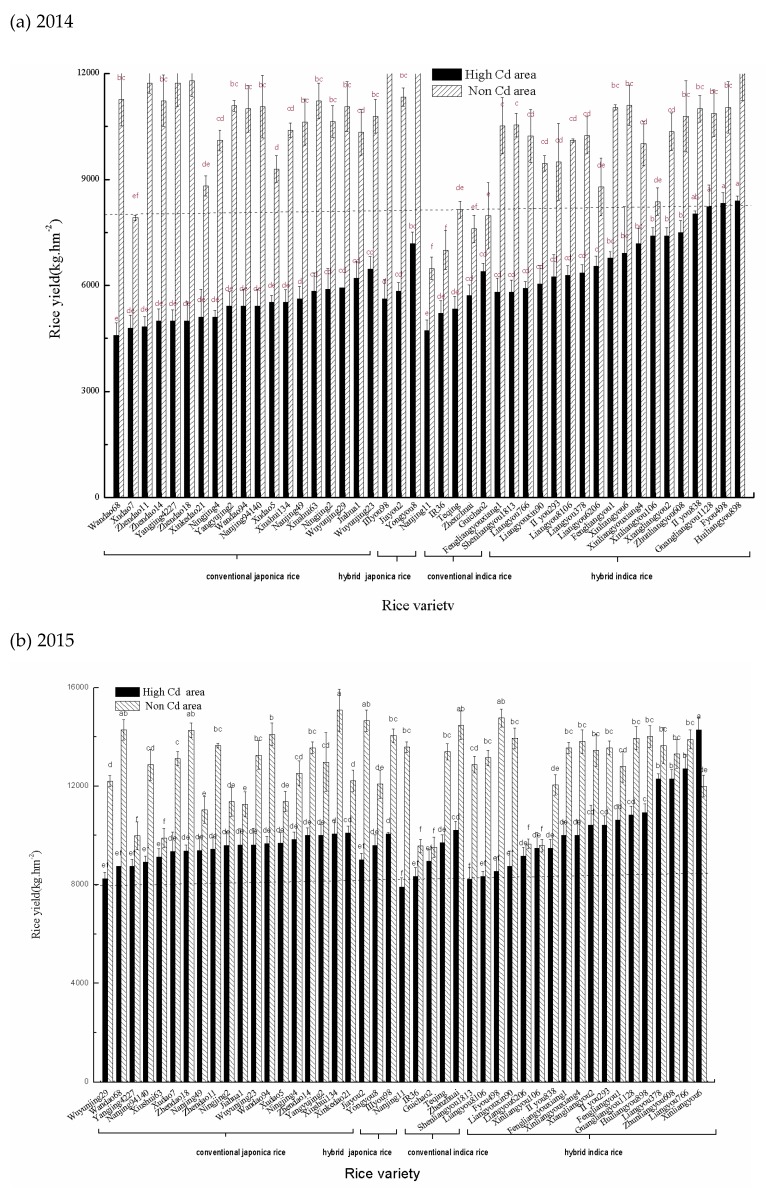
Rice yields in (**a**) 2014 rice season and (**b**) 2015 rice season. Different letters above columns indicate significant difference among varieties at 0.05 level.

Yields of the rice planted in the non-Cd area was significantly higher than those in the high-Cd area (Figure 2). In 2014, rice yields in the high-Cd area were 4583–8396 kg·hm^−2^ with an average of 6086 kg·hm^−2^ and a variance index at 16.7%. The varieties including Huiliangyou 898, Fyou 498, Guangliangyou 1128, II you 838, Zhunliangyou 608, Xiangliangyou 2, Xinliangyou 106, Xinliangyouxiang 4, Yongyou 8, Xinliangyou 6, and Fengliangyou 1 produced relatively higher yields. 

In the non-Cd farmland, rice yields were 6479–13042 kg·hm^−2^ with an average of 10265 kg·hm^−2^ and the variance index of 13.9%. The high yield varieties included Huiliangyou 898, Yongyou 8, Xinliangyou 6, Xiushui 63, Jiayou 2, III you 98, Zhendao 18, Yangjing 4227, Zhendao 14, Zhendao 11, etc. 

In 2015, rice yields in the high-Cd area were 7917–14292 kg·hm^−2^ with an average of 9782 kg·hm^−2^ and the variance index of 12.6%. The high yield varieties included Xinliangyou 6, Liangyou 766, Liangyou 378, Zhunliangyou 608, Huiliangyou 898, Guangliangyou 1128, Fengliangyou 1, Xiangliangyou 2, II you 293, Zhenzhuai, Xinkedao 21, etc.

In the non-Cd area, rice yields were 9515–18089 kg·hm^−2^ with an average of 12765 kg·hm^−2^ and the variance index of 12.1%. The high yield rice varieties included Zhendao 18, Jiayou 2, Zhenzhuai, Xiushui 134, Wandao 94, Wandao 68, Fyou 498, etc.

### 3.3. Relationship between Rice Grain Cd Accumulation and Rice Yields

The results showed that there was a significant negative relation between grain Cd content and yields. The relationship between Cd content in rice grains and rice yields in 2014 rice season is

(1) $$y\;=10128.8-4074.3\;e^{\left(-\frac{0.0000014}{x^{7}}\right)}{}^{\;}\;\;(\mathrm{correlation}\;\mathrm{coefficient}:\;0.822)$$

and the relationship between Cd content in rice grains and rice yields in the 2015 rice season is

y = 9679/(1 − 0.295)*e^(-5.06x)^ (correlation coefficient: 0.765). (2)

**Figure 3:**
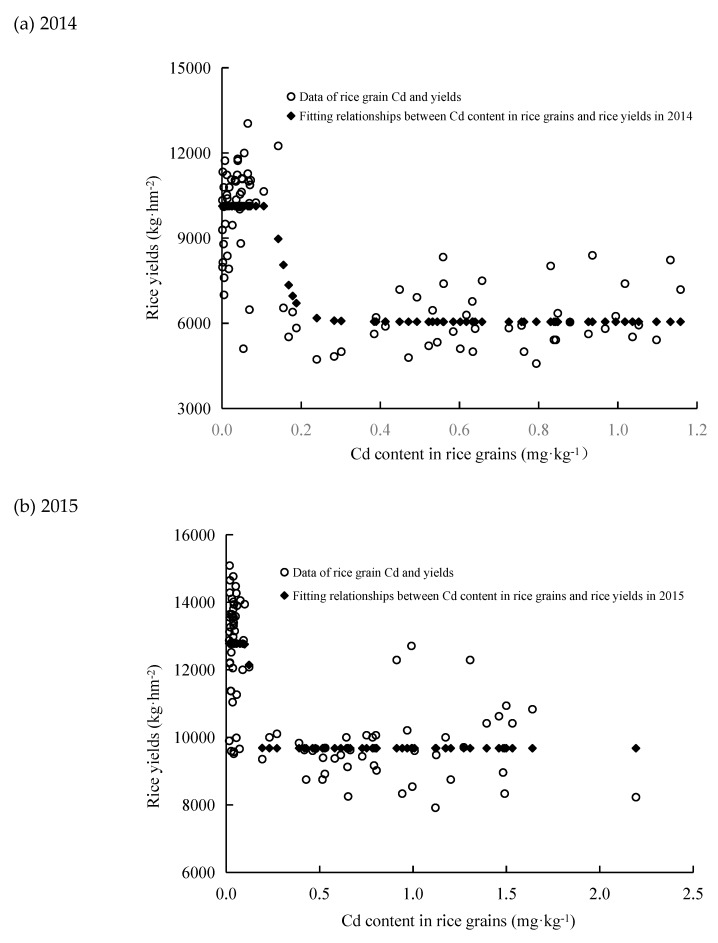
Relationships between Cd content in rice grains and rice yields: (**a**) 2014, (**b**) 2015.

These results may provide a theoretical basis for the selection of rice varieties with low cadmium accumulation and high yields. Rice yields seemed stabilized when Cd concentration in rice grain increased from 0 to 0.12 mg·kg^−1^ in 2014 and increased from 0 to 0.11 mg·kg^−1^ in 2015. Rice yields were reduced significantly when rice grain Cd accumulation increased from 0.12 to 0.24 mg·kg^−1^ in 2014 and increased from 0.11 to 0.19 mg·kg^−1^ in 2015. With further increasing in Cd content in rice, the rice yields seemed changed significantly (Figure 3).

### 3.4. Effects of Soil Amendments with a Mixture of Lime, Organic Fertilizers, and Porous Ceramics on Rice Cd Contents

Cd content of the rice from Cd heavily contaminated, lightly contaminated, and non-contaminated farmland in 2016 were presented in Table 2.

In 2016, we cultivated low Cd accumulating rice varieties in the high-Cd and low-Cd areas. In the low-Cd area, rice grain Cd content was 0.023–0.081 mg·kg^−1^, which met the national grain safety standard. Cd content of the rice planted in the low-Cd area was significantly lower than that planted in the high-Cd area. In the high-Cd area without soil amendments, rice Cd content was 0.11-0.81 mg·kg^−1^ with an average of 0.33 mg·kg^−1^. Cd content of rice varieties including Jiayou 2, Xudao 7, Xudao 5, Xiushui 63, Zhendao 11, and Ningjing 4 reached the national grain safety standard. In the high-Cd area, after using the mixture of lime, organic fertilizer, and porous ceramics, rice Cd content was reduced to 0.021–0.42 mg·kg^−1^ with an average of 0.16 mg·kg^−1^. The following 13 varieties rice were able to meet the national grain safety standard: Ningjing 4, Zhendao 11, Xiushui 63, Xudao 5, Xudao 7, Jiayou 2, Zhendao 14, Yangjing 4227, Zhendao 18, Xinliangyou 106, Xinkedao 21, Teqing, and Zhenzhuai. After the application of the amendment, the Cd content of rice grains decreased by 1.0–84.7% with an average of 52.6%. More importantly, these soil amendments significantly reduced Cd content of all rice varieties except Fengliangyouxiang 4, Nanjing 11, Wandao 68, Zhendao 14, and Ningjing 4.

### 3.5. Effects of a Mixture of Lime, Organic Fertilizer, and Porous Ceramics Nanomaterials on Soil Available Cd Content

Available Cd content of the soil from the high-Cd area, low-Cd area, and non-Cd area in 2016 were presented in Table 2.

The available Cd content of the soil from the low-Cd area was lower than that from the high-Cd area (Table 2). The soil available Cd content was 0.26–0.41 mg·kg^−1^ with an average of 0.36 mg·kg^−1^ in the low-Cd area while it was 0.60-1.48 mg·kg^−1^ (average of 1.04 mg·kg^−1^) in the high-Cd area without using soil amendments. After application of lime, organic fertilizers and porous ceramics in the high-Cd area, soil available Cd content reduced to 0.24–1.22 mg·kg^−1^ with an average of 0.74 mg·kg^−1^. Soil amendments were able to reduce soil available Cd content by 1.1–75.8% with an average of 27.7%. The reduction of available Cd content in soil was significant for rice varieties of Liangyou 8106, Zhenzhuai, Teqing, Fengliangyouxiang 1, Xinkedao 21, Yangjing 4227, Wandao 68, Zhendao 14, Jiayou 2, Xudao 7, Xudao 5, Xiushui 63, Zhendao 11, and Ningjing 4. At the same time, the significant reduction of Cd content in rice grains was found for rice varieties of Liangyou 8106, Zhenzhuai, Teqing, Xinkedao 21, Yangjing 4227, Jiayou 2, Xudao 7, Xudao 5, Xiushui 63, and Zhendao 11.

### 3.6. Effects of Lime, Organic Fertilizers, and Porous Ceramics on Rice Yields

Rice yields from the high-Cd area, low-Cd area, and non-Cd area in 2016 were presented in Table 3. 

Rice yields in the low-Cd area were significantly higher than those in the high-Cd area. Yields of the rice grown in the low-Cd area reached 10934–12384 kg·hm^−2^ with an average of 11697 kg·hm^−2^. In the High Cd area without using these amendments, rice yields were 7471–9778 kg·hm^−2^ with an average of 8699 kg·hm^−2^. High-yield rice varieties included Zhendao 11, Xudao 7, Jiayou 2, Xinliangyou 106, Liangyou 8106, and Xinkedao 21 with yields higher than 9100 kg·hm^−2^. At the same time, the rice varieties met the national food safety standard of grain Cd content included Jiayou 2, Xudao 7, and Zhendao 11. After using these amendments in the high-Cd area, rice yields increased to 7271–9370 kg·hm^−2^ with an average of 8516 kg·hm^−2^. The high-yield varieties included Xudao 7, Jiayou 2, Xinliangyou 106, Zhendao 11, Yangjing 4227, Liangyou 8106, and Teqing with yields higher than 8700 kg·hm^−2^. At the same time, grain Cd content of all rice varieties except Liangyou 8106 met the national food safety standard. After the application of the soil amendment, most of the rice varieties increased their yields but the increase was not significant. The rice yields of Jiayou 2 and Liangyou 766 were slightly reduced and the reduction was not significant. In general, the soil amendments did not have significant impacts on rice yields.

## 4. Discussion

### 4.1. Criteria for Low Accumulation Rice Varieties

Selection of low-cadmium rice varieties was the main strategy to prevent cadmium poisoning from entering the food chain and eventually entering humans. Plant growth, agronomic traits and low Cd uptake are major factors. Liu et al. proposed the following standards to evaluate low heavy metal accumulating varieties: a) The amount of heavy metal accumulated in plants should be low, or at least the contents in the edible parts must be lower than the national safety standards; b) Plants with a strong tolerance to heavy metals can grow as normal plants on heavy metal contaminated soil without significantly reducing biomass production; c) The transport of heavy metal to above-ground tissues is minimum. Heavy metals must be accumulated mainly in roots and the transportation index should be below 1.0; and d) Heavy metal contents in plants should be lower than the soil level and the enrichment index should be lower than 1.0 [17]. Based on the present study, we suggested the following criteria: the low metal accumulation in the edible parts should be persistent for at least three growth seasons. 

The single contamination index was recommended by China Green Food Development Center. This method was also used in the analyses of heavy metal contamination [18]. The present study used the Secondary Standards in GB 15618-1995 Environmental Quality Standard for soil. The farmland had soil Pi value of Cd of 0.87, 1.30, and 6.1, for non-contaminated, lightly contaminated, and heavily contaminated soil, respectively. 

### 4.2. Mechanisms of Low Accumulation of Cd in Crops

The distribution of heavy metals in crops may be divided into four compartments, including chloroplasts and plastids, cell walls, soluble parts, and organelles [19]. The accumulation of heavy metals in plants is related to their physiological processes of absorption, transport, and detoxification. Crops such as rice can reduce the transport of heavy metals through several defense mechanisms including combination with cell walls, active efflux and through plasma membranes. Several metal detoxification mechanisms, such as chelating and metal thioredin detoxification, could also be used to reduce the accumulation of heavy metals and their toxicity to the crops [20]. Studies on rice, Arabidopsis, and other organisms showed that metal transporters, ABC transporters, and natural resistance-associated macrophage protein transporters were involved in cadmium stress response. There were nine metal transporters found in rice. OsHMA9-OsHMA4 for the monovalent cations such as Ag/Cu; OsHMA3-OsHMA1 for the divalent cations including Cd/Co/Zn/Pb [21]. The cell walls of crop roots are mainly composed of polysaccharides and proteins, which provide negative charge points on the surface and limit the transmembrane transport of metal ions. They are the first barrier to protect protoplast from metal toxicity [22]. Vacuoles occupy 90% of the cell volume [23] and contain abundant peptides and organic acids [24]. Vacuolar storage is the main process to limit the transfer of heavy metals in plants [20]. After accumulation of heavy metals, rice and other crops can still grow normally depending on detoxification mechanisms in crops, mainly through chelating metal ions with high affinity ligands [25]. Potential ligands for heavy metals include SH group peptides, such as plant chelating peptide (PC), glutathione (GSH) and small genes encode proteins rich in cysteine - metallothionein (MT) [20]. Cai et al. reported that exogenously added reduced glutathione improved the cadmium tolerance of rice [26]. Hu et al. (2009), also showed that glutathione S-transferase and reduced glutathione in rice played an important role in cadmium passivation and detoxification [27]. The glutamine synthetase also participated in the detoxification of metal ions mediated by the plant chelating peptides [28]. 

### 4.3. Cd Grain Accumulation among Different Rice Varieties

Several previous studies revealed the differences in Cd uptake and accumulation among rice genotypes, which could be determined by genes (G), environmental factors (E), or their interactions [29,30]. Studies also showed that the cadmium content of seven rice types was in the order of *Xenopus laevis*, conventional japonica rice, conventional late japonica rice, two-line late rice, three-line late rice, conventional early Indica, and special rice [31]. However, Xu et al. reported that Cd contents of polished rice differed among various varieties, which could not be distinguished between hybrids and conventional rice in general [15]. A large-scale survey of 20 provinces in the four rice growing regions in China indicated that Cd content in 712 samples of rice varied from <0.001 to 0.74 mg·kg^−1^ [32]. They also showed that there were significant genetic variations in Cd accumulation capacity of the 45 rice varieties. Conventional Japonica and Indica rice varieties of these 45 rice cultivars accumulated less Cd than hybrid ones, which was in agreement with the previous study. It has been reported that Cd accumulation capacity of brown rice decreased in the order of Indica hybrids> Japonica hybrids > conventional Indica rice > conventional Japonica rice [33]. In present study, there was also significant variation in Cd accumulation capacity among rice varieties. Conventional varieties accumulated less Cd than hybrid varieties in high-Cd area. Therefore, screened rice varieties may be safely produced in Cd lightly polluted soil. 

### 4.4. Effects of Cd on Crop Growth and Development

Several studies reported that heavy metals in plants, when reaching to a threshold level, became toxic to plants causing disruption in metabolic pathways, slowed crop growth and lowered yields by the following mechanisms [34]. Cd causes DNA damage in rice. Cd^2+^ can cause abnormal DNA methylation, which affects gene viability, interferes with the synthesis of enzymes and proteins, and fundamentally causes damage to crop growth and development [35]. Low concentration of Cd^2+^ promoted the respiration rate of rice leaves. However, higher concentrations of Cd^2+^ can cause a significant decrease in the content of Photosystem II, Photosystem I and light-harvesting pigment-protein complexes in leaves, thereby inhibiting the photosynthetic rate. In addition, Cd^2+^ can also inhibit the mutual transformation of photosynthetic products in leaves, interfere with the output of leaf photosynthetic products and inhibit the input of photosynthetic products, resulting in the accumulation of small molecules of sugar in the leaves, which leads to the feedback inhibition of photosynthesis. Cd^2+^ can cause a decrease in the water-soluble protein content of rice roots and leaves, leading to inhibition of protein hydrolysis and synthesis. Cd^2+^ can also inhibit amylase and MDH activity and isozyme expression in various organs, preventing the tricarboxylic acid cycle [36,37].

The present study showed that there were some differences in yields among rice varieties. Rice Cd content had a significant negative correlation with yields. Grain yields in the low-Cd area were significantly higher than that in the high-Cd area.

### 4.5. Effect of Soil Amendment on the Accumulation of Cd in Crops

Organic matters have large specific surface area and a large number of functional groups. Humic acids could form a chelate complex with soil Cd. These interactions reduced the mobility of Cd and thus reduce its uptake into plants [38]. A large number of studies showed that organic fertilizers effectively increased crop yields [39,40]. Zhao et al. and Li et al. reported that when organic fertilizers were used in place of inorganic fertilizers, rice yields increased significantly [41,42]. 

The characteristics of the porous ceramic nanomaterials included high molecular recognition capability, sand particle structure, and optimized slow release. These materials were extremely effective in soil remediation against heavy metal contamination. The field experiments in Hunan Province of China showed that, after using these soil amendment in rice fields, water soluble Cd in soils was reduced from 322 ppb to 0.89 ppb resulting in a 99% absorption rate. The elimination rate of soil available Cd was 85%. The porous ceramics nanomaterials were applied in rice fields resulting in a 61% reduction in soil Cd content [13]. Lime also changed soil CEC, soil pH, soil redox status, and soil microbial communities. All of these factors affected soil capacity in chelation, precipitation, and adsorption of heavy metals [43,44].

Several studies indicated that soil available Cd content had a significant negative correlation with soil pH. Soil pH increased after amendment with lime, which reduced soil available Cd content and then less Cd uptake into plants [45]. Gu et al. reported that limes, when combined with other soil amendments were more effective than it was used alone in soil remediation [46]. Other studies confirmed that lime and Ca compounds were able to bind up to 95–99% of Cd, Pb, Cr, and As and other heavy metals [47,48].

In the present study, the application of lime, organic fertilizer, and porous ceramic nanomaterials as soil amendments in the high-Cd area was able to reduce the soil available Cd content and the Cd content in rice grain with little effects on rice yields. The cadmium content of grains met the national food safety standards and guaranteed the safe consumption of rice. This integrated remediation approach proved effective in improving remediation efficiency. However, the dosage and the types of soil amendment should be determined according to the species of heavy metals, the level of contamination, and type of soil. 

## 5. Conclusions

There was significant variation in Cd accumulation capacity among rice varieties. In the non-Cd area, Cd content in all rice grains reached the national food safety standard. In the high-Cd area conventional varieties accumulated less Cd than hybrid varieties. The following 19 rice varieties (Ningjing 4, Zhendao 11, Xiushui 63, Xudao 5, Xudao 7, Jiayou 2, Zhendao 14, Wandao 68, Yangjing 4227, Zhendao 18, Nanjing 11, Fengliangyouxiang 1, Liangyou 6206, Xinliangyou 106, Xinkedao 21, Teqing, Liangyou 766, Zhenzhuai, Liangyou 8106) showed the characteristics of low accumulation of cadmium. Among them, five varieties exhibited high yields and low Cd accumulating traits: Zhenzhuai, Liangyou 766, Xinkedao 21, Ningjing 4, and Xinliangyou 106. There was no significant difference in yields among rice varieties. Cd content in rice grains had a significant negative correlation with yields. 

Application of lime, organic fertilizer, and porous ceramic nanomaterials as soil amendments in the high-Cd area was able to reduce the soil available Cd content and the Cd content in rice grains with little effect on rice yields. The low-Cd accumulating varieties selected in this study were all within the national grain safety standard in the Cd lightly contaminated fields. In the high-Cd area without soil amendments, rice Cd content of six varieties (Ningjing 4, Zhendao 11, Xiushui 63, Xudao 5, Xudao 7, Jiayou 2) were within the national grain safety standard limits. After the application of soil amendements, the following 13 varieties rice were able to meet the national grain safety standard (Ningjing 4, Zhendao 11, Xiushui 63, Xudao 5, Xudao 7, Jiayou 2, Zhendao 14, Wandao 68, Yangjing 4227, Zhendao 18, Xinliangyou 106, Xinkedao 21, Teqing). Therefore, the current study implies that proper agronomic management with selection of rice varieties and soil amendments is essential in controlling Cd accumulation in rice grains.

## Figures and Tables

**Table 1:** Selected soil physicochemical properties.

Degree of Pollution	Soil Available Cd	Soil Total Cd	pH	CEC	Alkaline Hydrolytic Nitrogen	Soil Available Phosphorus	Rapidly Available Potassium	Organic Matters
	mg·kg^−1^	mg·kg^−1^		cmol·kg^−1^	mg·kg^−1^	mg·kg^−1^	mg·kg^−1^	g·kg^−1^
Non-Cd area	0.172	0.261	6.51	14.79	123.57	19.78	81.25	25.71
Low-Cd area	0.361	0.389	5.93	13.56	133.35	19.25	136.67	22.47
High-Cd area	1.36	1.83	5.03	12.95	120.27	19.15	74.32	23.66

**Table 2:** Cd content in rice grains and bioavailable Cd content in soils with different treatments.

Rice Varieties		High-Cd area		High-Cd Area + Soil Amendments		Low-Cd Area	
		Rice Grain Cd Content (mg·kg^−1^)	bioavailable Cd Content in Soils (mg·kg^−1^)	Rice Grain Cd Content (mg·kg^−1^)	Bioavailable Cd Content in Soils (mg·kg^−1^)	Rice Grain Cd Content (mg·kg^−1^)	Bioavailable Cd Content in Soils (mg·kg^−1^)
Indica rice variety	Liangyou 8106	0.81 ± 0.03a	0.96 ± 0.06a	0.42 ± 0.03b	0.47 ± 0.05b	0.039 ± 0.003c	0.38 ± 0.03c
	Zhenzhuai	0.56 ± 0.03a	1.12 ± 0.09a	0.14 ± 0.02b	0.64 ± 0.05b		
	Liangyou 766	0.56 ± 0.06a	0.67 ± 0.12a	0.26 ± 0.03b	0.59 ± 0.09a	0.081 ± 0.002c	0.41 ± 0.02b
	Teqing	0.53 ± 0.08a	1.32 ± 0.08a	0.18 ± 0.01b	0.86 ± 0.05b	0.039 ± 0.003c	0.41 ± 0.01c
	Xinliangyou 106	0.48 ± 0.02a	1.07 ± 0.10a	0.17 ± 0.02b	0.99 ± 0.14a	0.023 ± 0.004c	0.41 ± 0.08b
	Liangyou 6206	0.38 ± 0.05a	0.92 ± 0.05a	0.22 ± 0.02b	0.81 ± 0.07a		
	Fengliangyouxiang1	0.35 ± 0.01a	1.21 ± 0.05a	0.27 ± 0.07a	1.07 ± 0.07b	0.042 ± 0.003b	0.34 ± 0.03c
	Nanjing 11	0.29 ± 0.04a	0.60 ± 0.04a	0.23 ± 0.02a	0.57 ± 0.04a		
Japonica rice variety	Xinkedao 21	0.52 ± 0.05a	1.11 ± 0.09a	0.13 ± 0.01b	0.73 ± 0.07b		
	Zhendao 18	0.26 ± 0.02a	1.24 ± 0.07a	0.09 ± 0.02b	1.22 ± 0.09a	0.029 ± 0.004c	0.36 ± 0.06b
	Yangjing 4227	0.25 ± 0.02a	1.48 ± 0.06a	0.15 ± 0.02b	0.85 ± 0.09b	0.023 ± 0.003c	0.41 ± 0.01c
	Wandao 68	0.24 ± 0.04a	1.25 ± 0.05a	0.23 ± 0.02a	1.03 ± 0.09b		
	Zhendao 14	0.22 ± 0.03a	1.01 ± 0.13a	0.14 ± 0.04a	0.24 ± 0.03b		
	Jiayou 2	0.17 ± 0.03a	1.11 ± 0.09a	0.11 ± 0.01b	0.38 ± 0.03b		
	Xudao 7	0.14 ± 0.02a	1.08 ± 0.06a	0.03 ± 0.01b	0.62 ± 0.03b	0.038 ± 0.003b	0.35 ± 0.05c
	Xudao 5	0.14 ± 0.03a	0.96 ± 0.08a	0.02 ± 0.005b	0.66 ± 0.06b	0.045 ± 0.002b	0.26 ± 0.04c
	Xiushui 63	0.14 ± 0.01a	1.08 ± 0.18a	0.02 ± 0.005b	0.75 ± 0.09b	0.034 ± 0.002c	0.41 ± 0.04c
	Zhendao 11	0.12 ± 0.02a	0.60 ± 0.08a	0.05 ± 0.004b	0.59 ± 0.04a		
	Ningjing 4	0.11 ± 0.02a	0.86 ± 0.10a	0.11 ± 0.02a	0.65 ± 0.07b	0.060 ± 0.003b	0.40 ± 0.04c

Different lowercase letters (a, b, c) following data in each row of the table indicate significant difference among treatments at 0.05 level.

**Table 3:** Rice yields with different treatments.

Rice Variety		Rice Yields (kg·hm^−2^)		
		High-Cd Area	High-Cd Area + Soil Amendment	Low-Cd Area
Indica rice variety	Liangyou 8106	9520 ± 24a	8967 ± 17a	11339 ± 32b
	Zhenzhuai	8453 ± 24a	8259 ± 27a	
	Liangyou 766	8225 ± 13a	7661 ± 11b	11379 ± 18c
	Teqing	8685 ± 23a	8759 ± 23a	11772 ± 35b
	Xinliangyou 106	9331 ± 20a	9370 ± 22a	12384 ± 20b
	Liangyou 6206	8817 ± 24a	8535 ± 24a	
	Fengliangyouxiang 1	8841 ± 5a	8850 ± 12a	12169 ± 10b
	Nanjing 11	8789 ± 34a	8880 ± 27a	
Japonic rice variety	Xinkedao 21	8975 ± 38a	8520 ± 12a	
	Zhendao 18	8441 ± 34a	8483 ± 11a	12088 ± 12b
	Yangjing 4227	8834 ± 18a	8891 ± 6a	11474 ± 23b
	Wandao 68	8730 ± 35a	8667 ± 13a	
	Zhendao 14	7694 ± 14a	7661 ± 21a	
	Jiayou 2	9778 ± 28a	9004 ± 11b	
	Xudao 7	9330 ± 28a	9231 ± 11a	12003 ± 16b
	Xudao 5	7490 ± 14a	7271 ± 12a	10934 ± 10b
	Xiushui 63	8442 ± 19a	8036 ± 41a	11276 ± 20b
	Zhendao 11	9165 ± 31a	8849 ± 13a	
	Ningjing 4	7471 ± 28a	7436 ± 21a	11441 ± 23b

Different lowercase letters (a, b, c) following data in each row of the table indicate significant difference among treatments at 0.05 level.
